# Development of deoxidation process for off-grade titanium sponge using magnesium metal with wire mesh strainer type of crucible

**DOI:** 10.1038/s41598-023-50765-2

**Published:** 2024-01-04

**Authors:** Sung-Hun Park, Hyeong-Jun Jeoung, Tae-Hyuk Lee, Ho-Sang Sohn, Jungshin Kang

**Affiliations:** 1 Research Institute of Energy and Resources, Seoul National University, 1 Gwanak-ro, Gwanak-gu, Seoul, 08826 Republic of Korea; 2 Department of Materials Science and Engineering, Seoul National University, 1 Gwanak-ro, Gwanak-gu, Seoul, 08826 Republic of Korea; 3 Resources Utilization Research Division, Korea Institute of Geoscience and Mineral Resources, 124 Gwahak-ro Yuseong-gu, Daejeon, 34132 Republic of Korea; 4 School of Materials Science and Engineering, Kyungpook National University, 80 Daehak-ro, Buk-gu, Daegu, 41566 Republic of Korea; 5 Department of Energy Resources Engineering, Seoul National University, 1 Gwanak-ro, Gwanak-gu, Seoul, 08826 Republic of Korea

**Keywords:** Metals and alloys, Process chemistry

## Abstract

In this study, the deoxidation process for off-grade titanium (Ti) sponge using magnesium (Mg) metal with a wire mesh strainer type of crucible was developed. Ti hydride (TiH_2_) feedstock, which was prepared by hydrogenating off-grade Ti sponge, was deoxidized using Mg in a molten magnesium chloride–potassium chloride salt at 933 K under an argon and 20% hydrogen (H_2_) mixed gas atmosphere. After deoxidation, the residual Mg-containing salt was separated in situ from the crucible to investigate the feasibility of minimizing salt loss during the leaching and production of pure TiH_2_. The results showed that the presence of residual Mg-containing salt inside the crucible strongly influenced whether a mixture of Ti and TiH_2_ or pure TiH_2_ was produced. When the salt was not sufficiently separated, a mixture of Ti and TiH_2_ was obtained and its oxygen (O) concentration was 0.121 mass% under certain conditions. Meanwhile, pure TiH_2_ was obtained by increasing the H_2_ gas flow rate during deoxidation. Therefore, these results demonstrate that the decrease of O concentration to below 0.180 mass% and the minimal loss of the salt are feasible.

## Introduction

Titanium (Ti) is used in aerospace, biomedical, and chemical processing applications because it and its alloys exhibit excellent properties such as high specific strength, corrosion resistance, and biocompatibility. Accordingly, the demand for Ti, particularly for aerospace applications, continues to increase^1–3^. However, the global production of Ti is lower than that of iron (Fe) and aluminum (Al)^4^. The main reasons for the low production volume of Ti are the low productivity and high processing costs of conventional Ti production processes^5^.

In conventional Ti production, the Kroll process, several factors are responsible for its low productivity and high processing cost, such as a batch-type operation and the long processing time^6,7^. Notably, off-grade Ti sponge is generated during the Kroll process^8,9^. It is estimated that 10–20% of the total production volume of Ti sponge^10^. This indicates that the high processing costs can be reduced by developing an efficient utilization method for off-grade Ti sponge. When off-grade Ti sponge can be utilized in Ti ingot production, it not only reduces the high processing costs of Ti but also compensates for the low Ti production volume. In addition, this can decrease carbon dioxide emissions by 95.4% because upgrading low-grade Ti ore and the Kroll process are not required^11^. Therefore, the recycling of off-grade Ti sponge is important.

The main challenge associated with the recycling of off-grade Ti sponge is the removal of major impurities such as oxygen (O) and Fe. These impurities negatively affect the mechanical properties of the final product and are almost impossible to remove from Ti using conventional remelting techniques. As a result, in modern industrial practice, off-grade Ti sponge is cheaply used as an additive in the steel industry. However, when the O concentration in off-grade Ti sponge can be lowered to below 0.180 mass%, which is the American Society for Testing Materials (ASTM) criteria for Grade 1 Ti^12^, off-grade Ti sponge can be utilized in Ti alloy production. Although Fe cannot be removed directly from off-grade Ti sponge, it can be used as a *β*-stabilizer in *β*-Ti alloys^13,14^. Therefore, to recycle off-grade Ti sponge for use in Ti production, a deoxidation process to decrease its O concentration to below 0.180 mass% is necessary.

Numerous methods for deoxidation of Ti using various reducing agents have been proposed. Due to the high affinity of O to Ti, most reducing agents used in the proposed deoxidation processes are limited to rare-earth elements, such as holmium (Ho)^15^, yttrium (Y)^16,17^, and lanthanum (La)^18^, calcium (Ca)^19–29^, and electrons^30–32^. However, magnesium (Mg), which is the cheapest among the metallic reducing agents, is not suitable as a reducing agent for deoxidation of Ti. This is because the O concentration in Ti by Mg/magnesium oxide (MgO) equilibrium at 1200 K is approximately 2 mass% in the standard state^33^, which is significantly higher than the 0.180 mass%.

In recent years, several studies have been conducted on the deoxidation of Ti using Mg^34–40^ to increase its capability to decrease the O concentration in Ti. Some of these studies proposed the promising hydrogen (H) assisted Mg reduction process for the deoxidation of Ti using Mg^34–36^, in which the magnesiothermic reduction of Ti is conducted in a hydrogen gas (H_2_) atmosphere. In this process, the O concentration in Ti is decreased to 0.0503 mass% when deoxidation is performed at 1023 K for 12 h in an H_2_ gas atmosphere^34^. This indicates that the O concentration in Ti can satisfy the ASTM criteria for Grade 1 Ti by the deoxidation even when Mg is used as the reducing agent under a high hydrogen chemical potential.

To develop a novel and efficient recycling process for off-grade Ti sponge, the deoxidation of off-grade Ti sponge using Mg in argon (Ar) and H_2_ mixed gas at 933–993 K was investigated by the authors^41,42^. To increase the activity of hydrogen (*a*_H_) in the reaction system and reaction rate, titanium hydride (TiH_2_) powder produced by hydrogenating an off-grade Ti sponge was used as the feedstock for deoxidation.

However, several drawbacks remain, such as the loss of residual Mg-containing salts during leaching and the production of a mixture of Ti and TiH_2_ instead of pure TiH_2_. The production of TiH_2_ is preferred to that of Ti, considering the sintering process after the deoxidation process. This is because the sintered compact using TiH_2_ powder has a higher sintered density than that using pure Ti powder, which improves the mechanical properties of the final Ti product^43,44^. However, in the authors’ previous studies^41,42^, a mixture of Ti and TiH_2_ was produced after the deoxidation. This was because TiH_2_ feedstock inside the salt was dehydrogenated during the deoxidation owing to the low hydrogen partial pressure ($p_{\text H_{2}}$) inside the salt. Therefore, the development of a method for the production of pure TiH_2_ is required even after the deoxidation.

In addition, salt loss was unavoidable because the salt was removed during subsequent leaching to recover the deoxidized product. It is worth noting that separating the residual Mg-containing salt from the deoxidized product before leaching can significantly reduce the amount of acid waste solution and the residual Mg-containing salt can be reused for deoxidation. Furthermore, reducing the amount of residual Mg-containing salt to be removed shortens the leaching time; thus, O contamination during leaching can be reduced. Therefore, to simultaneously overcome these drawbacks, the separation of residual Mg-containing salt during deoxidation is necessary.

**Figure 1:**
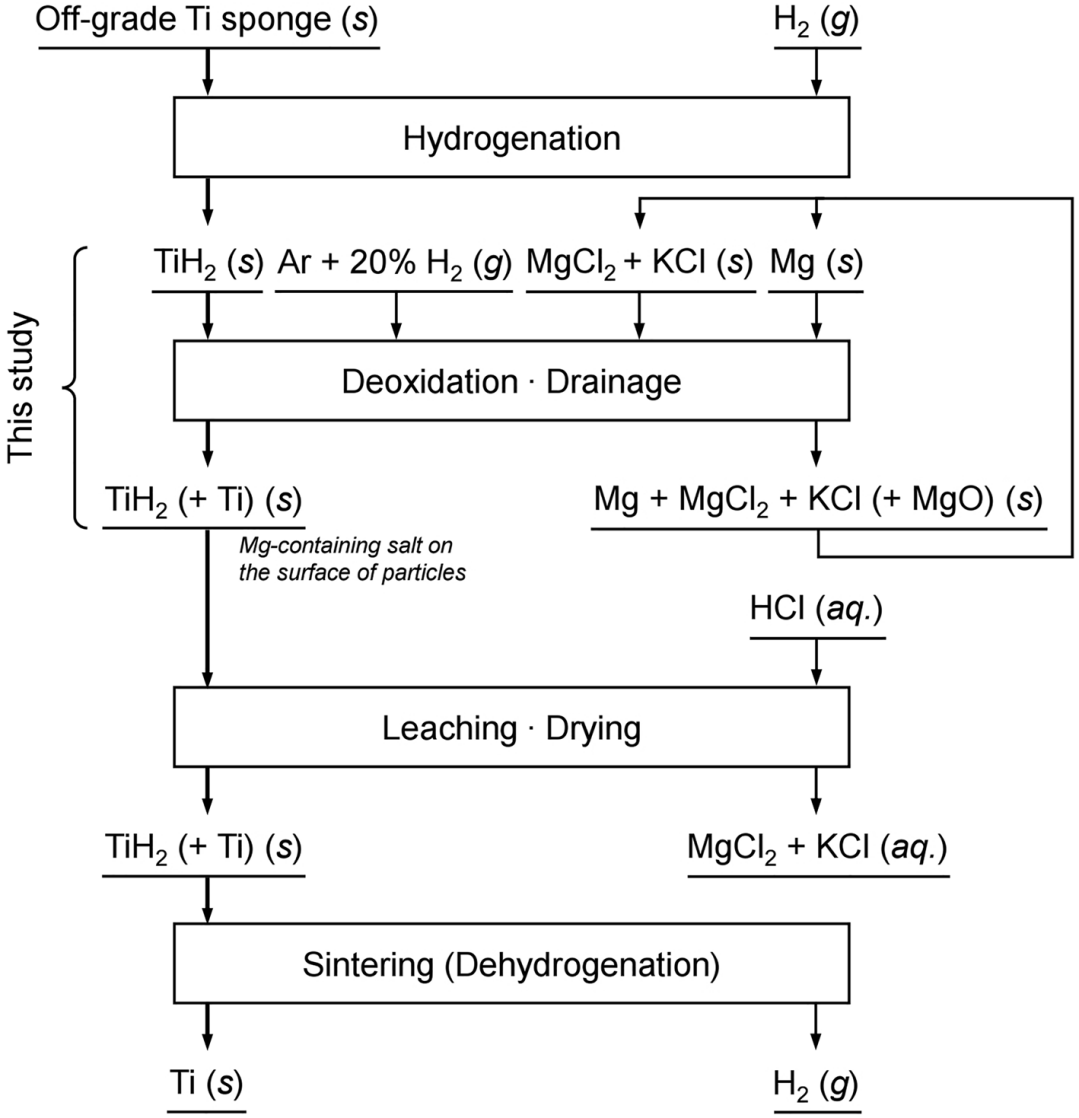
Flowchart of the deoxidation process for off-grade Ti sponge using Mg metal with a wire mesh strainer type of crucible in an Ar and 20% H_2_ mixed gas atmosphere.

In this study, a deoxidation process for off-grade Ti sponge was developed with the aim of decreasing the O concentration to below 0.180 mass%, minimizing the loss of residual Mg-containing salt, and producing pure TiH_2_. The influence of the reaction time on the O concentration in the residue was investigated. After deoxidation, the feasibility of separating the residual Mg-containing salt was investigated via in situ separation by lifting a wire mesh strainer type of crucible. Furthermore, the influences of the H_2_ gas flow rate, hydrogenation temperature, and draining time on the separation of the residual Mg-containing salt and the production of pure TiH_2_ were systematically investigated. Figure 1 shows a flowchart of the deoxidation process for off-grade Ti sponge using Mg with a wire mesh strainer type of crucible in an Ar and 20% H_2_ mixed gas atmosphere.

## Thermodynamic analysis

### Deoxidation of Ti using Mg in an Ar and H_2_ mixed gas atmosphere

**Figure 2:**
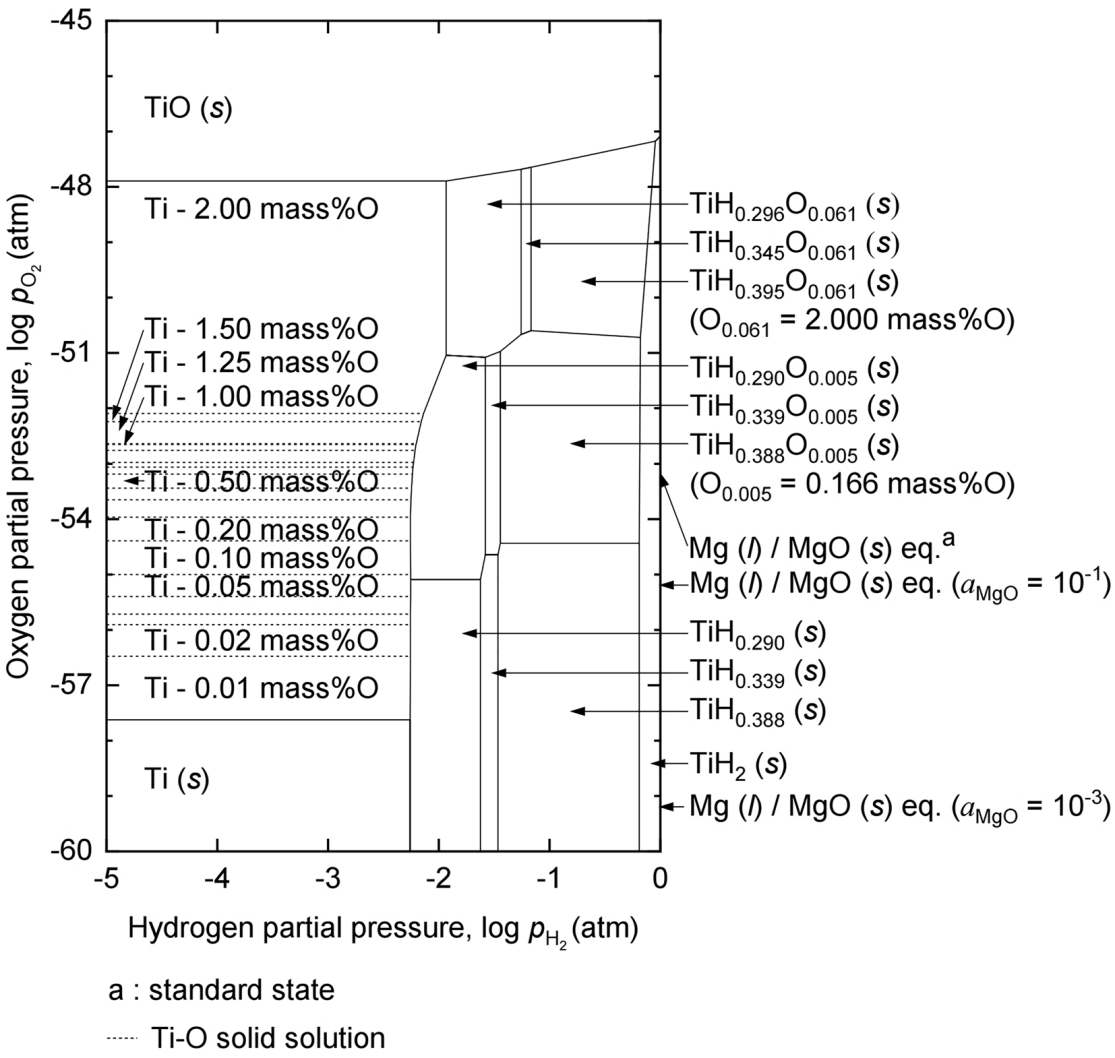
Chemical potential diagram of the Ti-O-H system at 973.15 K.

The chemical potential diagram of the Ti–O–H system at 973.15 K was plotted in Fig. 2 using the Chesta software^45^ and various thermodynamic data^34,46,47^. The ordinate denotes the oxygen partial pressure ($p_{\text O_{2}}$) and the abscissa denotes the $p_{\text H_{2}}$.

The stability regions of the Ti–O solid solutions are divided by dashed lines depending on the O concentration in Ti (*s*). The stability region of Ti-0.6 mass% O is located in the $p_{\text O_{2}}$ range of 6.5 × 10^−54^–8.7 × 10^−54^ atm and the $p_{\text H_{2}}$ range below 5.8 × 10^−3^–5.9 × 10^−3^ atm. The $p_{\text O_{2}}$ by the Mg (*l*)/MgO (*s*) equilibrium is 6.5 × 10^−54^ atm at 973.15 K under the standard state. This $p_{\text O_{2}}$ is located in the stability region of Ti-0.6 mass% O at $p_{\text H_{2}}$ below 5.8 × 10^−3^ atm. Therefore, when Ti is deoxidized using Mg at $p_{\text O_{2}}$ below 5.8 × 10^−3^ atm under a standard state, the O concentration in Ti will be 0.6 mass%, which is greater than 0.180 mass%. This indicates that the O concentration in Ti cannot be decreased to below 0.180 mass% when Ti is deoxidized using Mg without H_2_ gas under standard state.

However, there are two methods to decrease the O concentration in Ti to below 0.180 mass%, even when Mg is used as the reducing agent. One is to decrease the activity of MgO (*a*_MgO_). For example, when *a*_MgO_ is decreased to 0.1, the $p_{\text O_{2}}$ by Mg (*l*)/MgO (*s*) equilibrium decreases to 6.51 × 10^−56^ atm, as shown in Fig. 2. This $p_{\text O_{2}}$ is located in the stability region of Ti-0.05 mass% O even at $p_{\text H_{2}}$ below 5.6 × 10^−3^ atm. Therefore, although Mg is used as a reducing agent, the O concentration in Ti can be decreased to 0.05 mass% when the removal of the produced MgO during the deoxidation of Ti is maintained via chemical reactions or electrolysis^33,37,38,48^.

Another method is utilizing H_2_ to deoxidize Ti. As shown in Fig. 2, when $p_{\text O_{2}}$ is 6.51 × 10^−54^ atm determined by Mg (*l*)/MgO (*s*) equilibrium at 973.15 K, the stability regions of TiH_0.290_O_0.005_ (*s*), TiH_0.339_O_0.005_ (*s*), and TiH_0.388_O_0.005_ (*s*) are located at $p_{\text O_{2}}$ above 5.8 × 10^−3^ atm, 2.6 × 10^−2^ atm, and 3.6 × 10^−2^ atm, respectively. That is, when the $p_{\text H_{2}}$ is maintained above 5.8 × 10^−3^ atm and Mg is used as a reducing agent, the $p_{\text O_{2}}$ by Mg (*l*)/MgO (*s*) equilibrium is located in the stability regions of TiH_0.290_O_0.005_ (*s*), TiH_0.339_O_0.005_ (*s*), and TiH_0.388_O_0.005_ (*s*) with an O concentration of 0.166 mass%. Although only a few types of TiH_*x*_O_*y*_ are plotted owing to the lack of thermodynamic data^34,47^, this diagram demonstrates that the O concentration can be decreased to below 0.180 mass% by deoxidation using Mg when the $p_{\text H_{2}}$ is above 5.8 × 10^−3^ atm even under the standard state.

In this study, Mg was used as the reducing agent. In addition, TiH_2_ prepared in advance was used as the feedstock to increase the *a*_H_ in the reaction system. As shown in Fig. 1, TiH_2_ was produced by hydrogenation of off-grade Ti sponge before deoxidation. Furthermore, to maintain a high $p_{\text H_{2}}$ in the reaction system, an Ar and 20% H_2_ mixed gas was used during deoxidation and hydrogenation.

### TiH_2_ production in an Ar and H_2_ mixed gas atmosphere

To investigate the feasibility of TiH_2_ production, a thermodynamic analysis of the hydrogenation reaction is necessary. The production of TiH_2_ by the hydrogenation of Ti with H_2_ gas is described by Eq. (1)^46^. In addition, $p_{\text H_{2}}$ by Ti (*s*)/TiH_2_ (*s*) equilibrium can be expressed as shown in Eq. (2) using Eq. (1).

1 $${\text{Ti}}\;\left( s \right) + {\text{H}}_{{2}} \left( g \right) = {\text{TiH}}_{{2}} \;\left( s \right)$$

$$\Delta G^\circ_{{\text{r}}} = - {38}.{6}\;{\text{kJ} \cdot \text {mol}}^{-1}\;{\text{at}}\;{773}\;{\text{K}}$$

$$\Delta G^\circ_{{\text{r}}} = - {1}0.{4}\;{\text{kJ} \cdot \text {mol}}^{-1}\;{\text{at}}\;{973}\;{\text{K}}$$

2 $$p_{\text H_{2}} = {\text{exp }}(\Delta G^\circ_{{\text{r}}} /RT)$$

In Eqs. (1) and (2), $p_{\text H_{2}}$, ∆*G*°_r_, *R*, and *T* refer to the H_2_ partial pressure (atm), standard Gibbs free energy change for the reaction in Eq. (1) (kJ‧mol^−1^), universal gas constant (kJ‧mol^−1^‧K^−1^), and absolute temperature (K), respectively. As shown in Eq. (2), the $p_{\text H_{2}}$ by Ti (*s*)/TiH_2_ (*s*) equilibrium is a function of the temperature and was calculated to be 2.45 × 10^−3^ atm, 3.42 × 10^−2^ atm, and 1.27 × 10^−1^ atm at 773 K, 873 K, and 933 K, respectively (see Supplementary Fig. S1 online). This result indicates that the production of TiH_2_ (*s*) at 773–933 K in an Ar and 20% H_2_ mixed gas atmosphere is feasible.

In the previous study, although the TiH_2_ feedstock was deoxidized using Mg in an Ar and 20% H_2_ mixed gas atmosphere at 933 K, a mixture of Ti and TiH_2_ was produced instead of pure TiH_2_^41^. The TiH_2_ feedstock was deoxidized in a molten mixture of Mg and magnesium chloride (MgCl_2_)–potassium chloride (KCl). This indicates that the $p_{\text H_{2}}$ inside the molten Mg and salt was low, resulting in the dehydrogenation of TiH_2_ during deoxidation. As a result, the production of a mixture of Ti and TiH_2_ was unavoidable. It has also been reported that the diffusion of H_2_ gas to the Ti sub-hydride (TiH_*x*_ with *x* < 2) is impeded when MgCl_2_ covers the Ti sub-hydride powder^49^. Therefore, to increase the phase ratio of TiH_2_ in the mixture of Ti and TiH_2_, the $p_{\text H_{2}}$ inside the molten salt should be maintained at a high value during deoxidation or the molten salt should be separated during deoxidation enabling to expose of the deoxidized product to a high $p_{\text H_{2}}$.

In this study, a wire mesh strainer type of crucible was used for deoxidation. Deoxidation was performed by lowering the crucible, which contained the TiH_2_ feedstock prepared using off-grade Ti sponge, into the molten salt. After deoxidation was finished, the crucible was slightly raised above the surface of the molten salt and maintained for a certain period to separate the molten salt from the crucible and expose the feedstock to a high $p_{\text H_{2}}$.

## Methods

**Figure 3:**
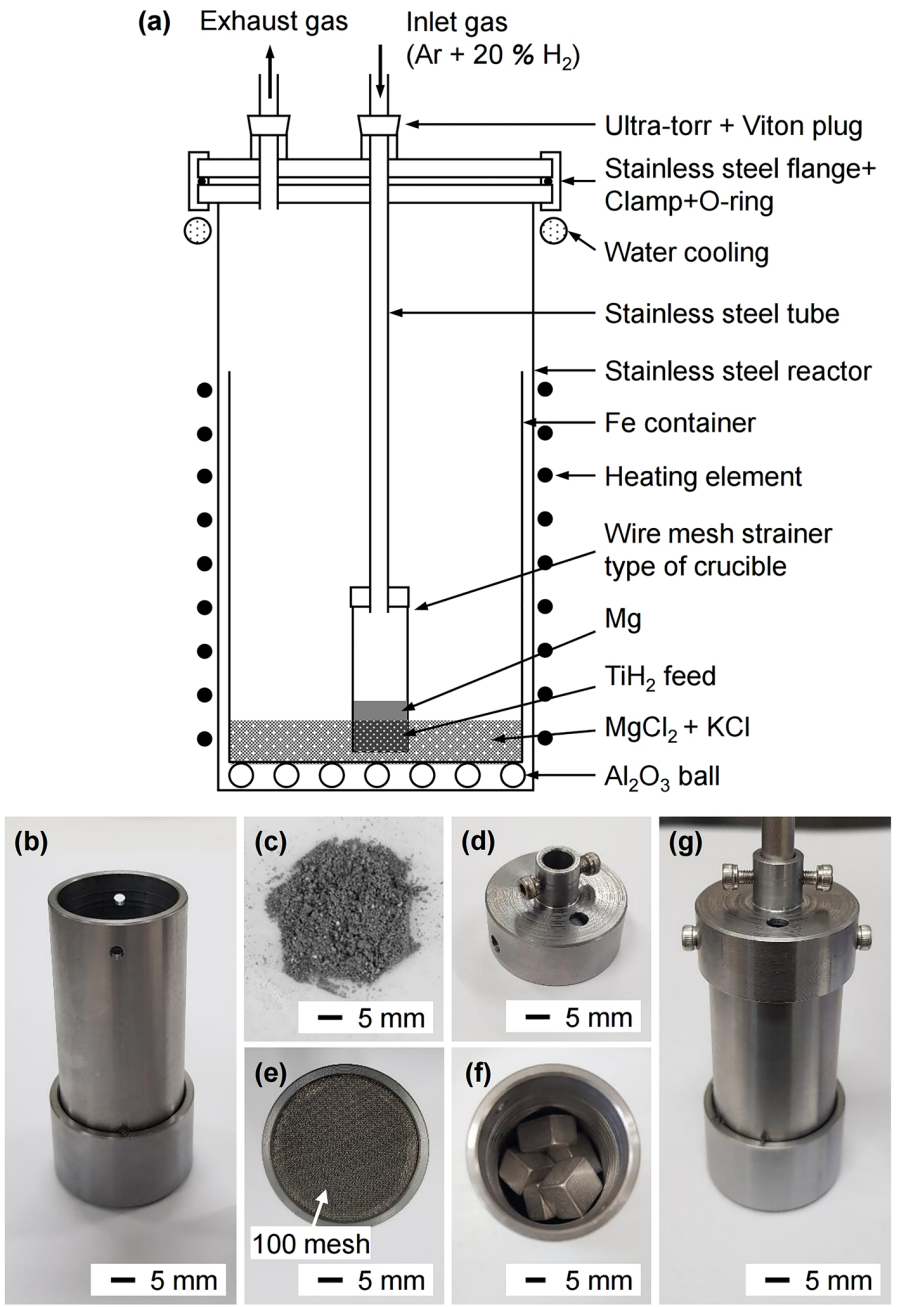
(**a**) Schematic of the experimental apparatus. Photographs of (**b**) the wire mesh strainer type of crucible, (**c**) TiH_2_ feedstock, (**d**) stainless steel cap, (**e**) Ti mesh (100 mesh) at the bottom of the crucible, (**f**) TiH_2_ feedstock and Mg inside the crucible, and (**g**) the crucible assembled with the stainless steel cap and tube.

Figure 3a shows the schematic of the experimental apparatus used in this study. In addition, Fig. 3b–g show photographs of parts of the Fe crucible assembly and samples used in the experiments. As shown in Fig. 3c, the TiH_2_ powder produced from off-grade Ti sponge was used as the feedstock for deoxidation. To prepare the TiH_2_ feedstock, off-grade Ti sponge (VSMPO-AVISMA Corporation) was hydrogenated at 973 K for 2 h in an H_2_ gas atmosphere at MTIG Co., Ltd. After hydrogenation, the TiH_2_ feedstock was pulverized using a mortar and pestle, followed by sieving. The particle size of TiH_2_ feedstock used for deoxidation was 150–300 μm.

Before deoxidation, the salt was dried and pre-melted to remove H_2_O from the chemical reagents. MgCl_2_ (anhydrous, purity > 97.0%, Wako Pure Chemical Corporation) and KCl (anhydrous, purity > 99.0%, KOJUNDO Chemical Laboratory Co., Ltd.) were dried at 453 K for 72 h in a vacuum oven (VOS-601SD, EYELA). Subsequently, the dried MgCl_2_ and KCl were placed inside a reactor, while being contained within an Fe container (outer diameter (*ϕ*) = 89 mm, thickness (*t*) = 2 mm, height (*h*) = 200 mm), and pre-melted at 933 K for 2 h in an Ar gas atmosphere.

After the salt was prepared, the TiH_2_ feedstock and Mg (cubic, width (*w*) = 10 mm, length (*l*) = 10 mm, height (*h*) = 10 mm, purity: 99.99%, RNDKOREA Corporation) were placed in a wire mesh strainer type (Ti mesh, mesh opening size = 100 mesh) of Fe crucible (outer diameter (*ϕ*) = 29 mm, thickness (*t*) = 2 mm, height (*h*) = 70 mm), as shown in Fig. 3f. The crucible was assembled with a stainless steel cap and stainless steel tube, as shown in Fig. 3g. The assembly was set up on the top flange of the reactor and positioned above the Fe container inside the reactor. The top flange and the reactor were fastened, and the reactor was placed in an electric furnace at 298 K.

The interior of the reactor was evacuated for 15 min at 298 K and then filled with an Ar and 20% H_2_ mixed gas to an internal pressure of 1 atm. The internal pressure of the reactor was maintained at 1 atm until the end of the experiment by constantly flowing mixed gas into the reactor. After controlling the atmosphere, the reactor was heated to 933 K. After 1 h at 933 K, the crucible was immersed in the molten salt by lowering it to 1 mm above the bottom of the Fe container for deoxidation of the TiH_2_ feedstock. After deoxygenation for the duration of 1–24 h, the crucible was lifted to 30 mm above the bottom of the Fe container to be positioned above the molten salt and held for 30 min or 3 h to drain the residual Mg-containing salt from the crucible through the Ti mesh. Subsequently, the crucible was lifted to 329 mm above the bottom of the Fe container, and the reactor was cooled to 298 K.

**Figure 4:**
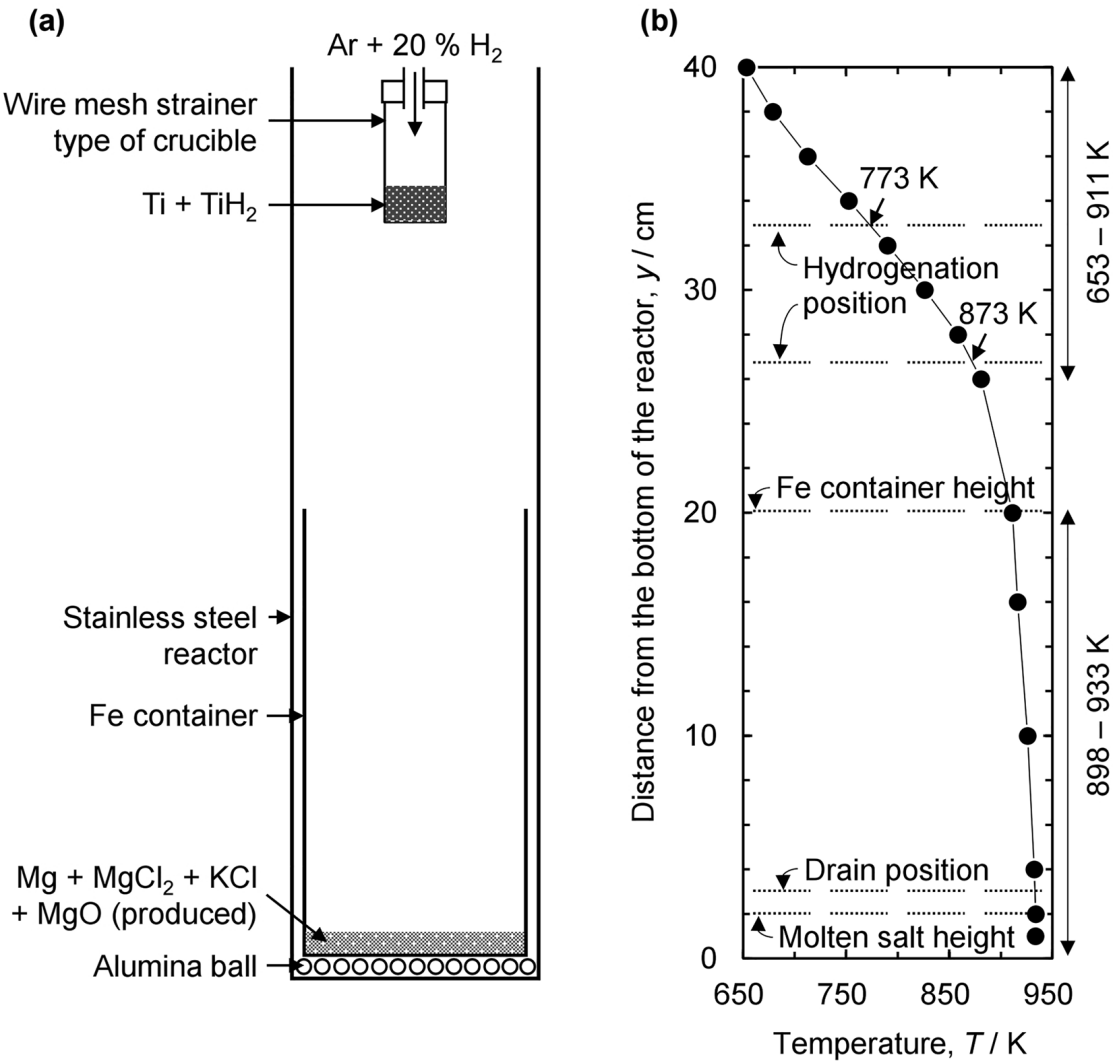
(**a**) Schematic of the interior of the reactor at the hydrogenation step after drainage of the residual Mg-containing salt and (**b**) temperature profile measured along the vertical distance from the bottom of the reactor at 933 K before deoxidation.

To hydrogenate deoxidized TiH_2_ feedstock, the temperature was held for 24 h after the crucible was lifted to 329 mm above the bottom of the Fe container where the temperature was 773 K, as shown in Fig. 4. In a different manner, after drainage of the residual Mg-containing salt, the crucible was lifted to 269 mm above the bottom of the Fe container where the temperature was 873 K and held for 24 h. The reactor was then cooled to 298 K.

**Figure 5:**
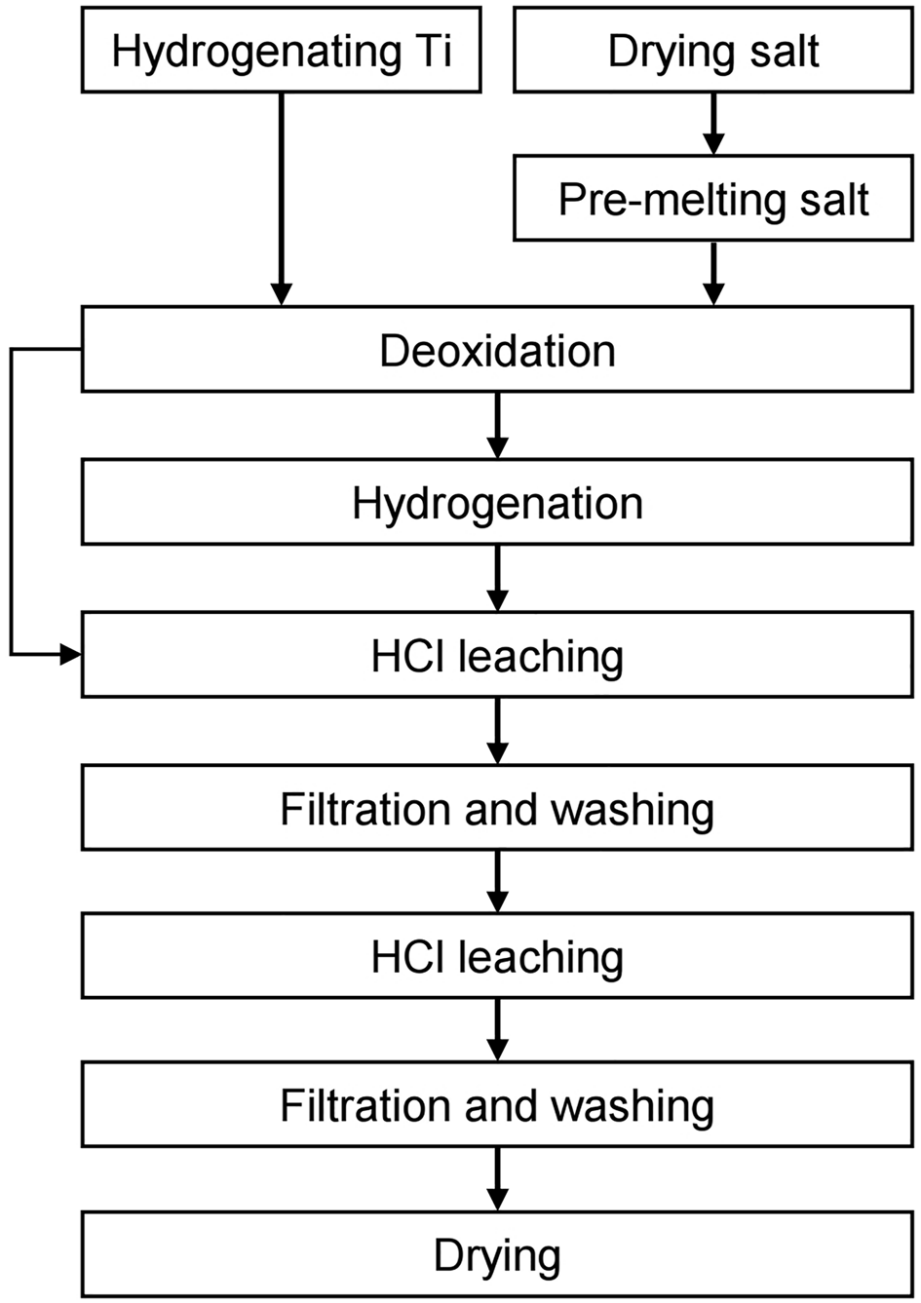
Flowchart of the experimental procedure for the deoxidation of off-grade Ti sponge using Mg metal and the production of TiH_2_ powder in an Ar + 20% H_2_ gas atmosphere.

After the experiments, the residual Mg, MgCl_2_, KCl, and MgO produced inside the crucible were removed by HCl leaching, as shown in Fig. 5. HCl leaching was performed using 1 L of 10% HCl solution at 298 K for 1 h without stirring and by blowing Ar gas into a jacketed reaction vessel. Subsequently, the residues were filtrated and washed with deionized (D.I.) water, followed by washing with acetone. The obtained residues were leached again using 1 L of 10% HCl solution at 298 K for 30 min with stirring at 150 rpm and by blowing Ar gas into a jacketed reaction vessel. After leaching, the residue was filtrated and washed with D.I. water, followed by washing with acetone.

The concentrations of O in the samples were analyzed using an N/O/H determinator (TCH600, LECO Corporation). The crystalline phases of the samples were identified using X-ray diffractometer (XRD: X'Pert MPD, PHILIPS, Cu-Kα radiation).

## Results and discussion

### Deoxidation of TiH_2_ feedstock

**Figure 6:**
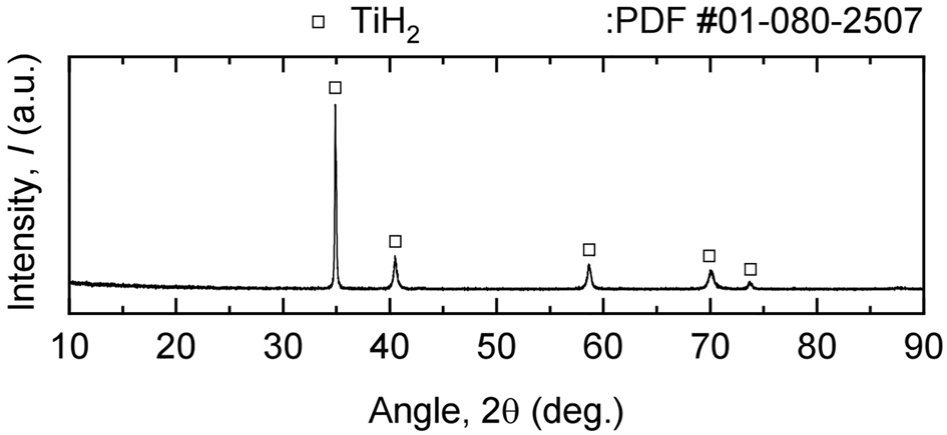
XRD analysis result of the TiH_2_ feedstock.

Figure 6 shows the TiH_2_, produced from off-grade Ti sponge by hydrogenation at 973 K for 2 h in an H_2_ gas atmosphere according to Eq. (1). The concentration of O in the TiH_2_ feedstock was 1.28 mass%, as listed in Table 1.

**Table 1:** Experimental conditions and results of the deoxidation of TiH_2_ feedstock.

Exp. no.^a^	Time for deoxidation, *t*_Deox_/h	Time for hydrogenation, *t*_Hydr_/h	Time for draining, *t*_Drain_/h	H_2_ gas flow rate		Temperature for hydrogenation, *T*_Hydr_/K	Mass fraction of MgCl_2_ in MgCl_2_–KCl, $f_{{\text {MgCl}}_2}$^b^	Concentration of O, *C*_O_ (mass%)^c^
				Deoxidation $f_{{\text H_2},{\text {Deox}}}$/sccm	Hydrogenation $f_{{\text H_2},{\text {Hydr}}}$/sccm			
TiH_2_ feed (using off-grade Ti sponge)								1.28
221005	1	–	0.5	300	–	–	0.75	0.218 ± 0.009
221013	3	–	0.5	300	–	–	0.75	0.158 ± 0.008
221019	6	–	0.5	300	–	–	0.75	0.140 ± 0.009
221028	12	–	0.5	300	–	–	0.75	0.132 ± 0.011
221110	24	–	0.5	300	–	–	0.75	0.121 ± 0.013
230105	6	24	3.0	300	300	773	0.55	0.149 ± 0.017
230127	6	24	3.0	300	500	773	0.75	0.154 ± 0.007
230203	6	24	3.0	300	500	873	0.75	0.158 ± 0.002
230111	6	24	3.0	500	500	773	0.75	0.194 ± 0.022

^a^Experimental conditions.

1) Weight of TiH_2_ feed, *w*_Feed_ = 10 g, Total weight of salt, *w*_Salt_ = 200 g, Weight of Mg, *w*_Mg_ = 10 g.

2) Temperature for deoxidation, *T*_Deox_ = 933 K, Atmosphere: Ar + 20% H_2_.

^b^Mass fraction of MgCl_2_ in MgCl_2_–KCl was calculated using following equation: weight of MgCl_2_/(weight of MgCl_2_ + weight of KCl).

^c^Determined by N/O/H analysis using TCH600 by LECO corporation.

In addition, Table 1 lists the experimental conditions for the deoxidation of TiH_2_ feedstock using Mg in an Ar and 20% H_2_ mixed gas atmosphere. In deoxidation, the mass ratios of Mg to feedstock (*r*_Mg/feedstock_) and salt to feedstock (*r*_salt/feedstock_) were maintained at 1 and 20, respectively. *r*_salt/feedstock_ and *r*_Mg/feedstock_ were calculated using Eqs. (3) and (4), respectively.

3 $$r_{{{\text{salt}}/{\text{feedstock}}}} = w_{{{\text{salt}}}} \left( {\text{g}} \right)/w_{{{\text{TiH}_2}\_{\text{feed}}}}(\text g)$$

4 $$r_{{{\text{Mg}}/{\text{feedstock}}}} = w_{{{\text{Mg}}}} \left( {\text{g}} \right)/w_{{{\text{TiH}_2}\_{\text{feed}}}}(\text g)$$

In Eqs. (3) and (4), *w*_salt_, $w_{{{\text{TiH}_2}\_{\text{feed}}}}$, and *w*_Mg_ refer to the weights of the salt, TiH_2_ feedstock, and Mg used, respectively.

**Figure 7:**
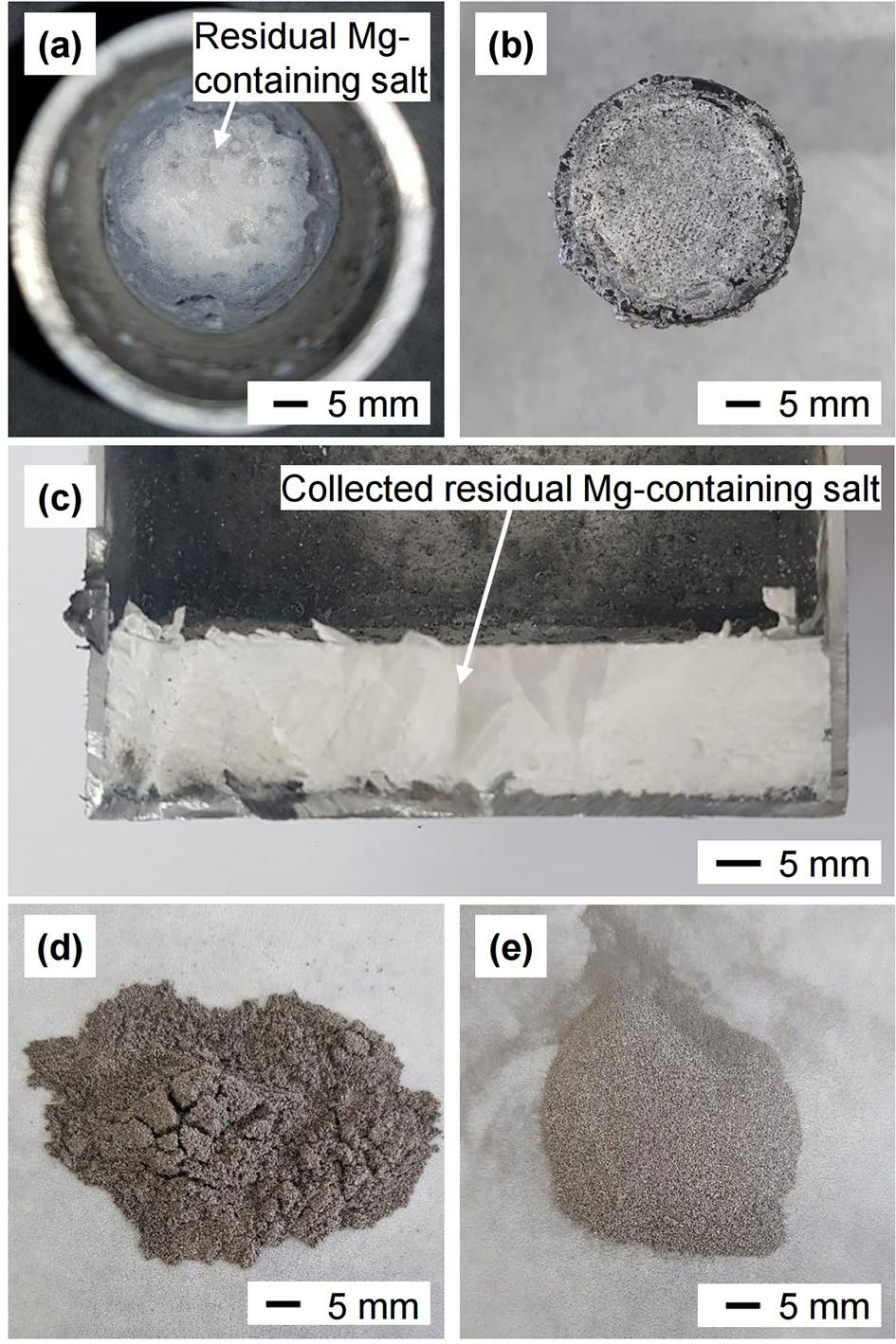
Representative photographs after the experiment: (**a**) residue inside the wire mesh strainer type of crucible, (**b**) residual Mg-containing salt under the Ti mesh at the bottom of the crucible, (**c**) cross-section of the salt collected inside the Fe container, (**d**) residue obtained after the first HCl leaching, and (**e**) residue obtained after the second HCl leaching.

Figure 7a–c show that the residual Mg-containing salt drained through the Ti mesh at the bottom of the crucible when the crucible was lifted to 30 mm above the bottom of the Fe container. The material remaining in the crucible was removed by HCl leaching according to the reactions shown in Eqs. (5) and (6)^50^. After HCl leaching, the deoxidized product was obtained as a fine powder, as shown in Fig. 7d,e.

5 $${\text{Mg}}\;\left( s \right) + {\text{2 HCl }}\left( {aq.} \right){\text{ = MgCl}}_{{2}} \;\left( {aq.} \right) + {\text{H}}_{{2}} \left( g \right)$$

$$\Delta G^\circ_{{\text{r}}} = - {463}.{1}\;{\text{kJ}\cdot{\text{mol}^{-1}}}\;{\text{at}}\;{298}\;{\text{K}}$$

6 $${\text{MgO}}\;\left( s \right) + {\text{2 HCl}}\;\left( {aq.} \right) = {\text{MgCl}}_{{2}} \left( {aq.} \right) + {\text{H}}_{{2}} {\text{O}}\;\left( l \right)$$

$$\Delta G^\circ_{{\text{r}}} = - {13}0.{9}\;{\text{kJ}\cdot{\text{mol}^{-1}}}\;{\text{at}}\;{298}\;{\text{K}}$$

As shown in Table 1, the O concentration in the feedstock of 1.28 mass% was decreased with an increasing deoxidation time. As a result, the lowest O concentration was 0.121 mass% when the TiH_2_ feedstock was deoxidized for 24 h.

As shown in Fig. 2, the equilibrium concentration of O in the deoxidized product was determined by the $p_{\text O_{2}}$, and $p_{\text O_{2}}$ was determined by the Mg (*l*)/MgO (*s*) equilibrium as a function of the activity of Mg (*a*_Mg_) and *a*_MgO_ in the reaction system. In this study, *a*_Mg_ was assumed to be unity during deoxidation. This is because, when the *r*_Mg/feedstock_ was maintained at 1, the amount of Mg used was 51 times larger than the amount required to reduce O in the TiH_2_ feedstock. The mass ratio of the Mg used to the Mg required was calculated using Eqs. (7)–(9). In addition, *a*_MgO_ was assumed to be unity because the solubility of MgO in MgCl_2_ at 1003 K and 1073 K were reported to be only 0.36 mol% and 0.63 mol%, respectively^51–53^. When considering the value of *r*_salt/feedstock_ and the amount of the MgO produced by complete deoxidation and MgCl_2_ used, the mol% of MgO in MgCl_2_ would be 0.51 mol%, which is greater than 0.36 mol%. In addition, the actual mol% of MgO in MgCl_2_ would be larger than 0.51 mol% when the generation of MgO by reacting MgCl_2_ with H_2_O absorbed to MgCl_2_ owing to its hygroscopic property^54,55^. Moreover, because the MgO solubility in MgCl_2_ decreases with a decreasing temperature, the MgO solubility in MgCl_2_ at 933 K is expected to be lower than that in MgCl_2_ at 1003 K, 0.36 mol%.

7 $${\text{Mg}}\;\left( l \right) + {\text{O}}\;\left( {s,\;{\text{in}}\;{\text{TiH}}_{{2}} \;{\text{feedstock}}} \right) = {\text{MgO}}\;\left( s \right)$$

8 $$w_{{{\text{Mg}}\;{\text{required}}}} = \left( {C_{{{\text{O}},\;{\text{in}}\;{\text{the}}\;{\text{feedstock}}}} \times w_{{{\text{TiH}_2}\_{\text{feed}}}} \left( {\text{g}} \right)/{1}00} \right) \times \left( {m_{{{\text{Mg}}}} /m_{{\text{O}}} } \right)$$

9 $$r_{{{\text{Mg}}\;{\text{used}}/{\text{Mg}}\;{\text{required}}}} = w_{{{\text{Mg}}}} \;\left( {\text{g}} \right)/w_{{\text{Mg required}}} \;\left( {\text{g}} \right)$$

In Eqs. (7)–(9), *w*_Mg required_, *C*_O, in the feedstock_, $w_{{{\text{TiH}_2}\_{\text{feed}}}}$, *m*_O_, *m*_Mg_, *r*_Mg used/Mg required_, and *w*_Mg_ refer to the calculated weight of Mg to deoxidize O in the TiH_2_ feedstock, initial O concentration of the feedstock used (mass%), weight of the TiH_2_ feedstock used (g), atomic mass of O (15.999 g·mol^−1^), atomic mass of Mg (24.305 g·mol^−1^), ratio of the amount of Mg used to Mg required, and weight of Mg used (g), respectively.

The thermodynamic analysis results presented in Fig. 2 show that Ti containing O of 0.166 mass% can be obtained under the standard state when the TiH_2_ is deoxidized using Mg at 973 K under a high hydrogen chemical potential. Although a detailed evaluation of the equilibrium concentration of O in Ti at 933 K was difficult owing to the lack of thermodynamic data, the experimental results, such as the deoxidized product obtained containing O of 0.121 mass%, demonstrated that the experimental results agreed with the thermodynamic analysis results. Consequently, these results show that the O concentration in the off-grade Ti sponge could be decreased to below 0.180 mass% by deoxidizing TiH_2_ using Mg at 933 K in an Ar and 20% H_2_ mixed gas atmosphere.

**Figure 8:**
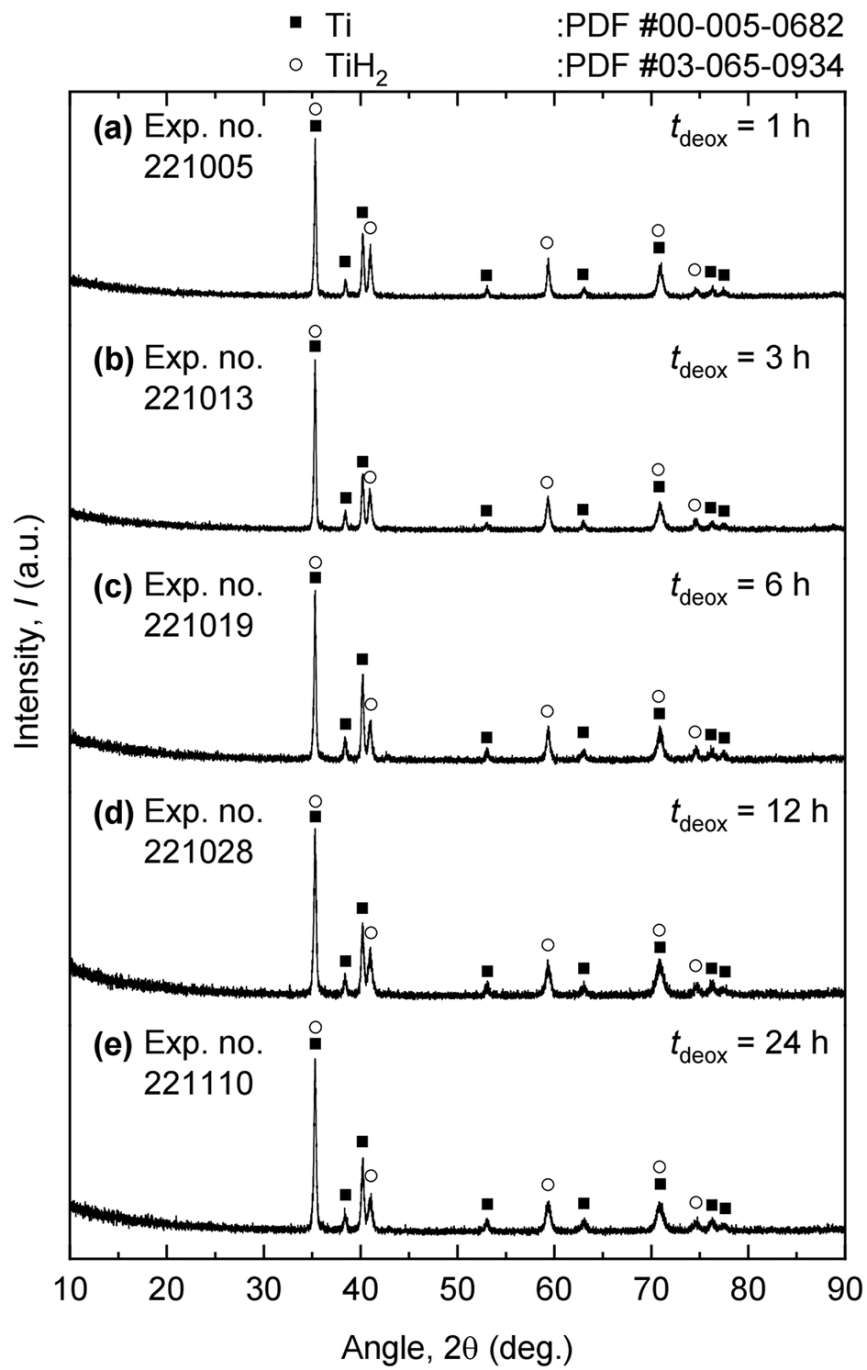
XRD analysis results of the residues obtained after the experiments at the deoxidation times of (**a**) 1 h, (**b**) 3 h, (**c**) 6 h, (**d**) 12 h, and (**e**) 24 h.

Figure 8 shows the XRD analysis results of the deoxidized product obtained after the experiments. The results showed that the obtained deoxidized product was a mixture of Ti and TiH_2_. This was because the residual Mg-containing salt was not drained enough to expose the deoxidized product to a high $p_{\text H_{2}}$, as shown in Fig. 7a. When the residual Mg-containing salt was not sufficiently drained, the deoxidized product was covered with the residual Mg-containing salt, placing the deoxidized product at low $p_{\text H_{2}}$. As a result, although high $p_{\text H_{2}}$ was maintained in the reaction system using an Ar and 20% H_2_ mixed gas, the generation of Ti was inevitable. Therefore, to obtain pure TiH_2_, the residual Mg-containing salt should be sufficiently drained from the crucible after deoxidation.

### Production of TiH_2_ by hydrogenation

To produce pure TiH_2_ by the hydrogenation of a mixture of Ti and TiH_2_ obtained after deoxidation, various attempts were made to sufficiently drain the residual Mg-containing salt from the crucible. As shown in Table 1, the H_2_ gas flow rates for deoxidation and hydrogenation, hydrogenation temperature, and mass fraction of MgCl_2_ in MgCl_2_–KCl were varied, whereas the times for deoxidation, hydrogenation, and draining were fixed at 6 h, 24 h, and 3 h, respectively.

**Figure 9:**
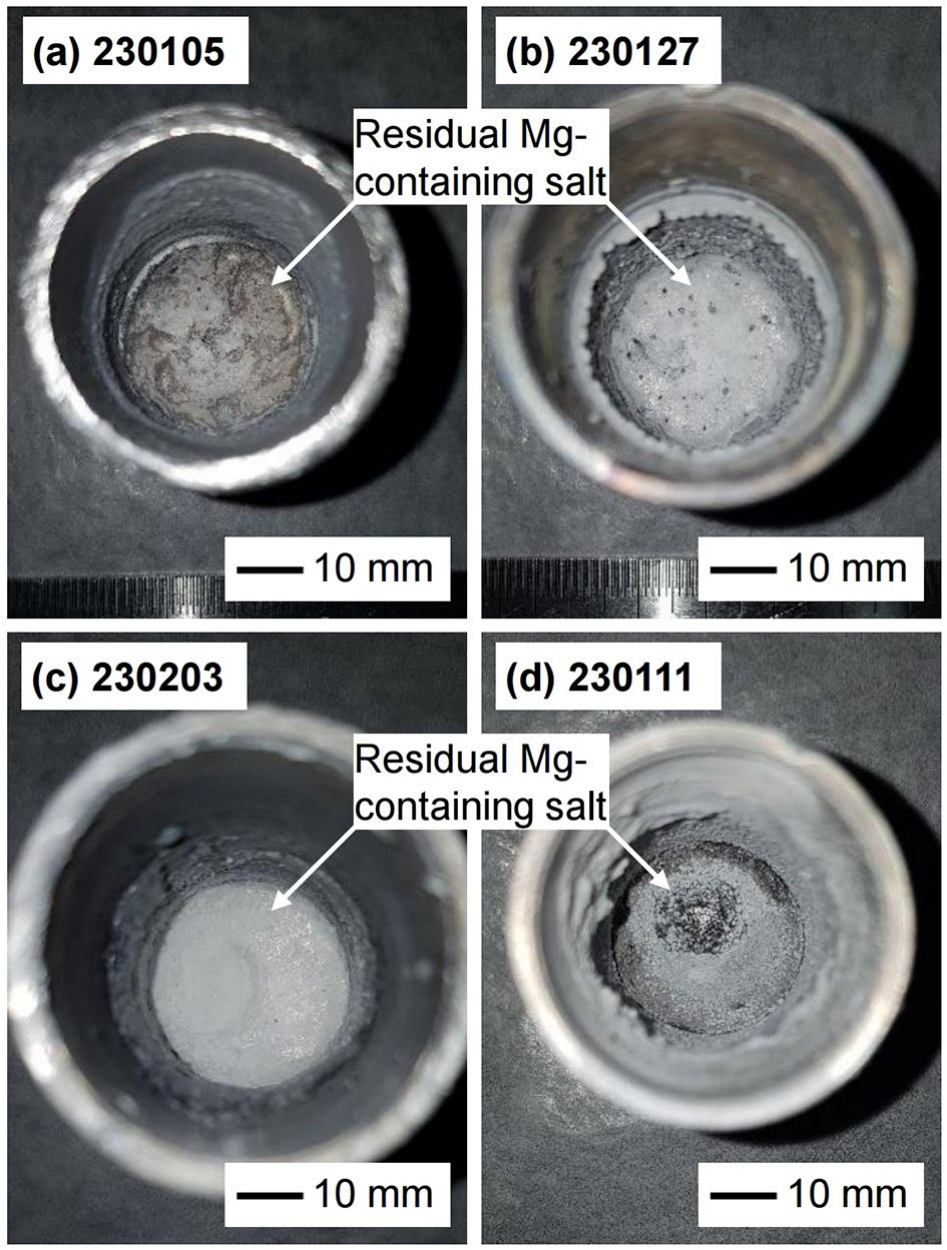
Photographs of the residue inside the wire mesh strainer type of crucible after the experiments using different $f_{{\text {MgCl}}_2}$, *T*_Hydr_, $f_{{\text H_2},{\text {Deox}}}$, and $f_{{\text H_2},{\text {Hydr}}}$ conditions: (**a**) 0.55, 773 K, 300 sccm, 300 sccm; (**b**) 0.75, 773 K, 300 sccm, 500 sccm; (**c**) 0.75, 873 K, 300 sccm, 500 sccm; and (**d**) 0.75, 773 K, 500 sccm, 500 sccm; $f_{{\text {MgCl}}_2}$, *T*_Hydr_, $f_{{\text H_2},{\text {Deox}}}$, and $f_{{\text H_2},{\text {Hydr}}}$ refer to the mass fraction of MgCl_2_ in MgCl_2_–KCl, temperature for hydrogenation, H_2_ gas flow rate during deoxidation, and H_2_ gas flow rate during hydrogenation, respectively.

Figure 9 shows photographs of the remains inside the crucible after deoxidation, followed by hydrogenation. As shown in Fig. 9a,d, the deoxidized product in powder form was exposed to the atmosphere, unlike the case shown in Fig. 9b,c. These results indicated that the influences of an increase in the H_2_ gas flow rate during hydrogenation and the hydrogenation temperature on the drainage of the residual Mg-containing salt were insignificant in these experiments, whereas a decrease in the mass fraction of MgCl_2_ in MgCl_2_–KCl and an increase in the H_2_ gas flow rate during deoxidation enabled further drainage of the salt from the crucible. This was because when the mass fraction of MgCl_2_ in MgCl_2_–KCl was decreased from 0.75 to 0.55, the melting point of the molten salt decreased from 844.3 K to 761.2 K (see Supplementary Fig. S2 online)^56^. This allowed the salt to exist as a liquid during hydrogenation at 773 K and to be further drained from the crucible during hydrogenation. In addition, when the H_2_ gas flow rate during deoxidation increased from 300 to 500 sccm, the outflow rate of the fluid through the Ti mesh also increased during drainage. Therefore, the residual Mg-containing salt was further drained from the crucible, thereby exposing the deoxidized product to high $p_{\text H_{2}}$ atmosphere.

**Figure 10:**
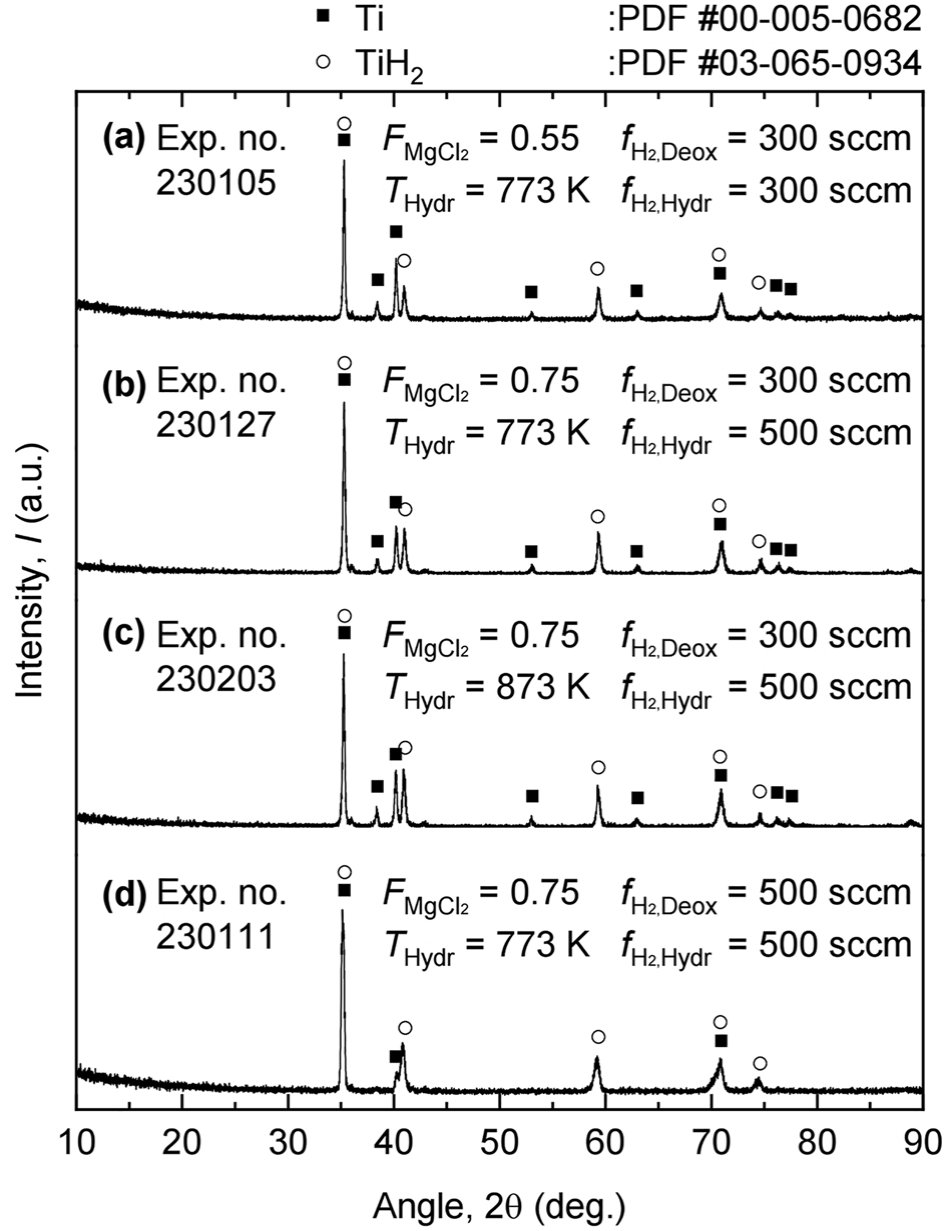
XRD analysis results of the residues obtained after the experiments using different $f_{{\text {MgCl}}_2}$, *T*_Hydr_, $f_{{\text H_2},{\text {Deox}}}$, and $f_{{\text H_2},{\text {Hydr}}}$ conditions: (**a**) 0.55, 773 K, 300 sccm, 300 sccm; (**b**) 0.75, 773 K, 300 sccm, 500 sccm; (**c**) 0.75, 873 K, 300 sccm, 500 sccm; and (**d**) 0.75, 773 K, 500 sccm, 500 sccm; $f_{{\text {MgCl}}_2}$, *T*_Hydr_, $f_{{\text H_2},{\text {Deox}}}$, and $f_{{\text H_2},{\text {Hydr}}}$ refer to the mass fraction of MgCl_2_ in MgCl_2_–KCl, temperature for hydrogenation, H_2_ gas flow rate during deoxidation, and H_2_ gas flow rate during hydrogenation, respectively.

Figure 10 shows the XRD analysis results of the deoxidized product obtained after HCl leaching. As shown in Fig. 10a–c, the obtained deoxidized products were identified as a mixture of Ti and TiH_2_. Unfortunately, although the deoxidized product was exposed to the atmosphere when the mass fraction of MgCl_2_ in MgCl_2_–KCl was decreased from 0.75 to 0.55, the deoxidized products obtained were identified as a mixture of Ti and TiH_2_. This indicated that the residual Mg-containing salt should be more drained from the crucible to produce pure TiH_2_. However, Fig. 10d shows that the peak of the Ti phase diminished, and that TiH_2_ was identified as the main phase in the obtained deoxidized product. This result indicates that the residual Mg-containing salt was sufficiently drained from the crucible when the H_2_ gas flow rate during deoxidation was increased from 300 to 500 sccm. Consequently, the deoxidized product was exposed to high $p_{\text H_{2}}$, which enabled its hydrogenation.

Meanwhile, the O concentrations in the deoxidized products obtained after deoxidation followed by hydrogenation are listed in Table 1. As shown in Table 1, most of the results demonstrate that the O concentration was decreased to below 0.180 mass%. However, when the H_2_ gas flow rate was increased from 300 to 500 sccm during deoxidation, the O concentration in the deoxidized product was 0.194 mass%, which is larger than 0.180 mass%. This was attributed to the use of a non-deoxidized Ar and 20% H_2_ mixed gas. It is considered that using a purified Ar and 20% H_2_ mixed gas is necessary to obtain pure TiH_2_ and decrease the O concentration to below 0.180 mass%.

However, the results demonstrated that a decrease in the O concentration to below 0.180 mass% by deoxidation using Mg at high $p_{\text H_{2}}$ was feasible, as expected from the thermodynamic analysis. Moreover, it was demonstrated that minimization of the loss of residual Mg-containing salt and the production of pure TiH_2_ by utilizing a wire mesh strainer type of crucible during deoxidation were feasible.

## Conclusions

The deoxidation process for off-grade Ti sponge using Mg with a wire mesh strainer type of crucible was developed with the aim of decreasing the O concentration to below 0.180 mass%, minimizing the loss of residual Mg-containing salt, and producing pure TiH_2_. After deoxidation using Mg in molten MgCl_2_–KCl salt at 933 K under an Ar and 20% H_2_ mixed gas atmosphere for 24 h, the O concentration in the TiH_2_ feedstock decreased from 1.28 mass% to 0.121 mass%. This result demonstrates that the O concentration can be efficiently decreased to below 0.180 mass% as expected from the results of the thermodynamic analysis. The production of either a mixture of Ti and TiH_2_ or pure TiH_2_ was significantly influenced by the drainage of the residual Mg-containing salt from the crucible. As a result, pure TiH_2_ was obtained by increasing the H_2_ gas flow rate during deoxidation. Therefore, it was demonstrated that the minimal loss of residual Mg-containing salt and the production of pure TiH_2_ by the deoxidation process developed in this study were feasible.

### Supplementary Information

Supplementary Figure S1.

Supplementary Figure S2.

## Data Availability

The datasets generated during and/or analysed during the current study are available from the corresponding author on reasonable request.

## References

[1] Coggins, A. Titanium Metal – Global supply and demand trends overview in *Titanium U. S. A. 2019 Conference* (2019).

[2] Pike, M. Titanium demand and trends in the commercial aero engine market. In *Titanium Virtual 2020 Conference* (2020).

[3] Carpenter, J. Boeing’s market outlook and titanium supply chain. In *Titanium U. S. A. 2019 Conference* (2019).

[4] U.S. Geological Survey. *Mineral Commodity Summaries* (2023).

[5] Dutta B, Froes FH, Qian M, Froes FH (2015). The additive manufacturing (AM) of titanium alloys. Titanium Powder Metallurgy: Science, Technology, and Applications.

[6] Kroll W (1940). The production of ductile Titanium. Trans. Electrochem. Soc..

[7] Kroll, W. Method for manufacturing Ti and alloys thereof. *US Patent 2205854* (1940).

[8] Takeda O, Ouchi T, Okabe TH (2020). Recent progress in titanium extraction and recycling. Metall. Mater. Trans. B.

[9] Sohn HS (2021). Current status of titanium recycling technology. Resour. Recycl..

[10] Marui Y, Kinoshita T, Takahashi K (2002). Development of a titanium material by utilizing off-grade titanium sponge. Honda R&D Tech. Rev..

[11] Corby, N. D. III. Titanium scrap trends. In *Titanium Virtual 2020 Conference* (2020).

[12] American Society for Testing and Materials, *Standard Specification for Titanium and Titanium Alloy Strip, Sheet, and Plate, B265–B06b* (ASTM International, 2006).

[13] Li CL (2019). Modeling hot deformation behavior of low-cost Ti-2Al-9.2Mo-2Fe beta titanium alloy using a deep neural network. J. Mater. Sci. Technol..

[14] Lütjering G, Williams JC (2007). Titanium, Engineering Materials and Processes.

[15] Kong L, Ouchi T, Okabe TH (2021). Deoxidation of Ti using Ho in HoCl_3_ flux and determination of thermodynamic data of HoOCl. J. Alloys Compd..

[16] Tanaka T, Ouchi T, Okabe TH (2020). Yttriothermic reduction of TiO_2_ in molten salts. Mater. Trans..

[17] Iizuka A, Ouchi T, Okabe TH (2020). Development of a new titanium powder sintering process with deoxidation reaction using yttrium metal. Mater. Trans..

[18] Tanaka T, Ouchi T, Okabe TH (2020). Lanthanothermic reduction of TiO_2_. Metall. Mater. Trans. B.

[19] Fisher, R. L. Deoxidation of titanium and similar metals using a deoxidant in a molten metal carrier. *US Patent 4923531A* (1990).

[20] Okabe TH, Suzuki RO, Oishi T, Ono K (1991). Thermodynamic properties of dilute titanium-oxygen solid solution in beta phase. Mater. Trans. JIM.

[21] Cho GH, Kim T, Chae J, Lim JW (2020). Preparing low-oxygen titanium powder by calcium reductant from titanium hydride. Adv. Powder Technol..

[22] Oh JM (2014). Preparation of low oxygen content alloy powder from Ti binary alloy scrap by hydrogenation–dehydrogenation and deoxidation process. J. Alloys Compd..

[23] Roh KM (2014). Comparison of deoxidation capability for preparation of low oxygen content powder from TiNi alloy scraps. Powder Technol..

[24] Okabe TH, Oishi T, Ono K (1992). Preparation and characterization of extra-low-oxygen titanium. J. Alloys Compd..

[25] Xia Y (2017). The effect of molten salt on oxygen removal from titanium and its alloys using calcium. J. Mater. Sci..

[26] Suzuki RO, Inoue S (2003). Calciothermic reduction of titanium oxide in molten CaCl_2_. Metall. Mater. Trans. B.

[27] Okabe TH, Oda T, Mitsuda Y (2004). Titanium powder production by preform reduction process (PRP). J. Alloys Compd..

[28] Xia Y (2018). Hydrogen enhanced thermodynamic properties and kinetics of calciothermic deoxygenation of titanium-oxygen solid solutions. Int. J. Hydrog. Energy.

[29] Li B (2021). The deep deoxygenation behavior of fine hydrogenated Ti alloy powders. JOM.

[30] Chen GZ, Fray DJ, Farthing TW (2000). Direct electrochemical reduction of titanium dioxide to titanium in molten calcium chloride. Nature.

[31] Chen GZ, Fray DJ, Farthing TW (2001). Cathodic deoxygenation of the alpha case on titanium and alloys in molten calcium chloride. Metall. Mater. Trans. B.

[32] Tripathy PK, Gauthier M, Fray DJ (2007). Electrochemical deoxidation of titanium foam in molten calcium chloride. Metall. Mater. Trans. B.

[33] Okabe TH, Hamanaka Y, Taninouchi YK (2016). Direct oxygen removal technique for recycling titanium using molten MgCl_2_ salt. Faraday Discuss..

[34] Zhang Y (2016). Thermodynamic destabilization of Ti-O solid solution by H_2_ and deoxygenation of Ti using Mg. J. Am. Chem. Soc..

[35] Xia Y (2017). Hydrogen assisted magnesiothermic reduction (HAMR) of commercial TiO_2_ to produce titanium powder with controlled morphology and particle Size. Mater. Trans..

[36] Dong Z (2020). Direct reduction of upgraded titania slag by magnesium for making low-oxygen containing titanium alloy hydride powder. Powder Technol..

[37] Zheng C, Ouchi T, Iizuka A, Taninouchi Y, Okabe TH (2019). Deoxidation of titanium using Mg as deoxidant in MgCl_2_-YCl_3_ flux. Metall. Mater. Trans. B.

[38] Jung JH, Lee SY, Park SH, Sohn HS (2021). Deoxidation of off-grade Ti scrap by molten Mg in YCl_3_-MgCl_2_ molten malt. Resour. Recycl..

[39] Kong L, Ouchi T, Okabe TH (2019). Direct deoxidation of Ti by Mg in MgCl_2_−HoCl_3_ flux. Mater. Trans..

[40] Tanaka T, Ouchi T, Okabe TH (2020). Magnesiothermic reduction of TiO_2_ assisted by LaCl_3_. J. Sustain. Metall..

[41] Lim KH, Jeoung HJ, Lee TH, Yi KW, Kang J (2022). Deoxidation of off-grade titanium sponge using magnesium metal in argon and hydrogen mixed gas atmosphere. Metall. Mater. Trans. B.

[42] Park SH (2023). Scale-up study of deoxidation of off-grade titanium sponge using magnesium metal under argon and hydrogen mixed gas atmosphere. J. Sustain. Metall..

[43] Robertson IM, Schaffer GB (2010). Comparison of sintering of Ti and Ti hydride powders. Powder Metall..

[44] Wang HT (2010). Ti and Ti alloy via sintering of TiH_2_. KEM.

[45] Hatada, N. *Chesta: Software for Creating Chemical Potential Diagrams, Version 3.4.3.2*. https://n-hatada.github.io/chesta.

[46] Barin I (1995). Thermochemical Data of Pure Substances.

[47] Mah, A. D., Kelley, K. K., Gellert, N. L., King, E. G. & O'Brien, C. J. *Thermodynamic Properties of Titanium-Oxygen Solutions and Compounds* (US Dept of the Interior, Bureau of Mines, 1957).

[48] Taninouchi Y, Hamanaka Y, Okabe TH (2016). Electrochemical deoxidation of titanium and its alloy using molten magnesium chloride. Metall. Mater. Trans. B.

[49] Park SH, Lee SY, Lee DH, Kang J, Sohn HS (2023). Production of titanium hydride powder from titanium tetrachloride using magnesium metal in hydrogen gas atmosphere. Mater. Trans..

[50] Outokumpu (2011). Chemistry for Windows, Version 7.11.

[51] Ito M, Morita K (2004). The solubility of MgO in molten MgCl_2_-CaCl_2_ salt. Mater. Trans..

[52] Zhang Y (2017). Kinetically enhanced metallothermic redox of TiO_2_ by Mg in molten salt. Chem. Eng. J..

[53] Boghosian S, Godø A, Mediaas H, Ravlo W, Østvold T (1991). Oxide complexes in alkali–alkaline–earth chloride melts. Acta Chem. Scand..

[54] Kang J, Okabe TH (2013). Removal of iron from titanium ore through selective chlorination using magnesium chloride. Mater. Trans..

[55] Kang J, Okabe TH (2014). Thermodynamic consideration of the removal of iron from titanium ore by selective chlorination. Metall. Mater. Trans. B.

[56] FactSage 8.2 Software. https://www.factsage.com.

