# Supplementary figures and images for: Development of deoxidation process for off-grade titanium sponge using magnesium metal with wire mesh strainer type of crucible
Source: Sci Rep. 2024 Jan 4;14:542. doi: 10.1038/s41598-023-50765-2 (PMC10766949; doi:10.1038/s41598-023-50765-2)

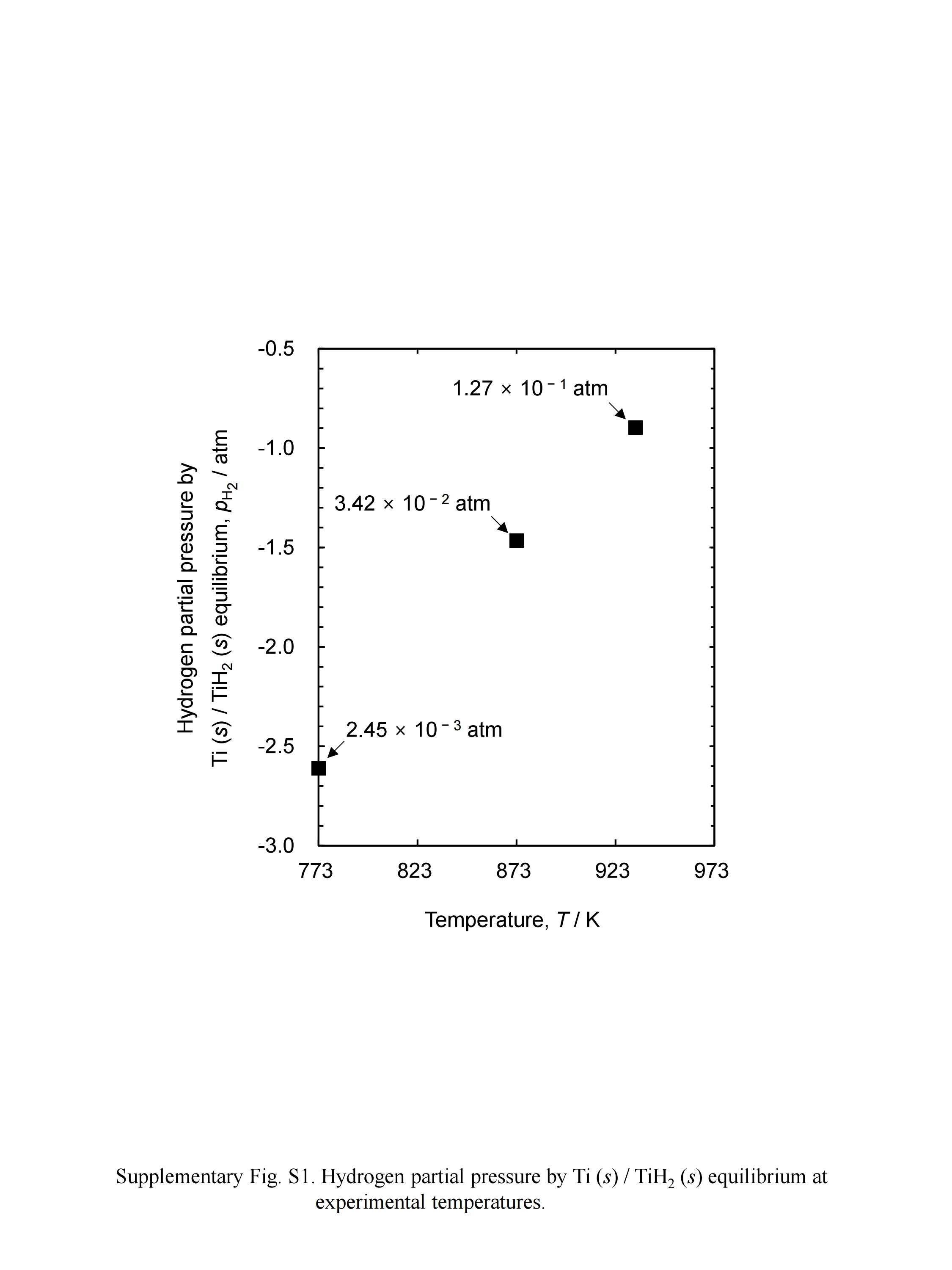

Supplement: Supplementary file 1 — Supplementary Figure S1. [file 41598_2023_50765_MOESM1_ESM.jpg]

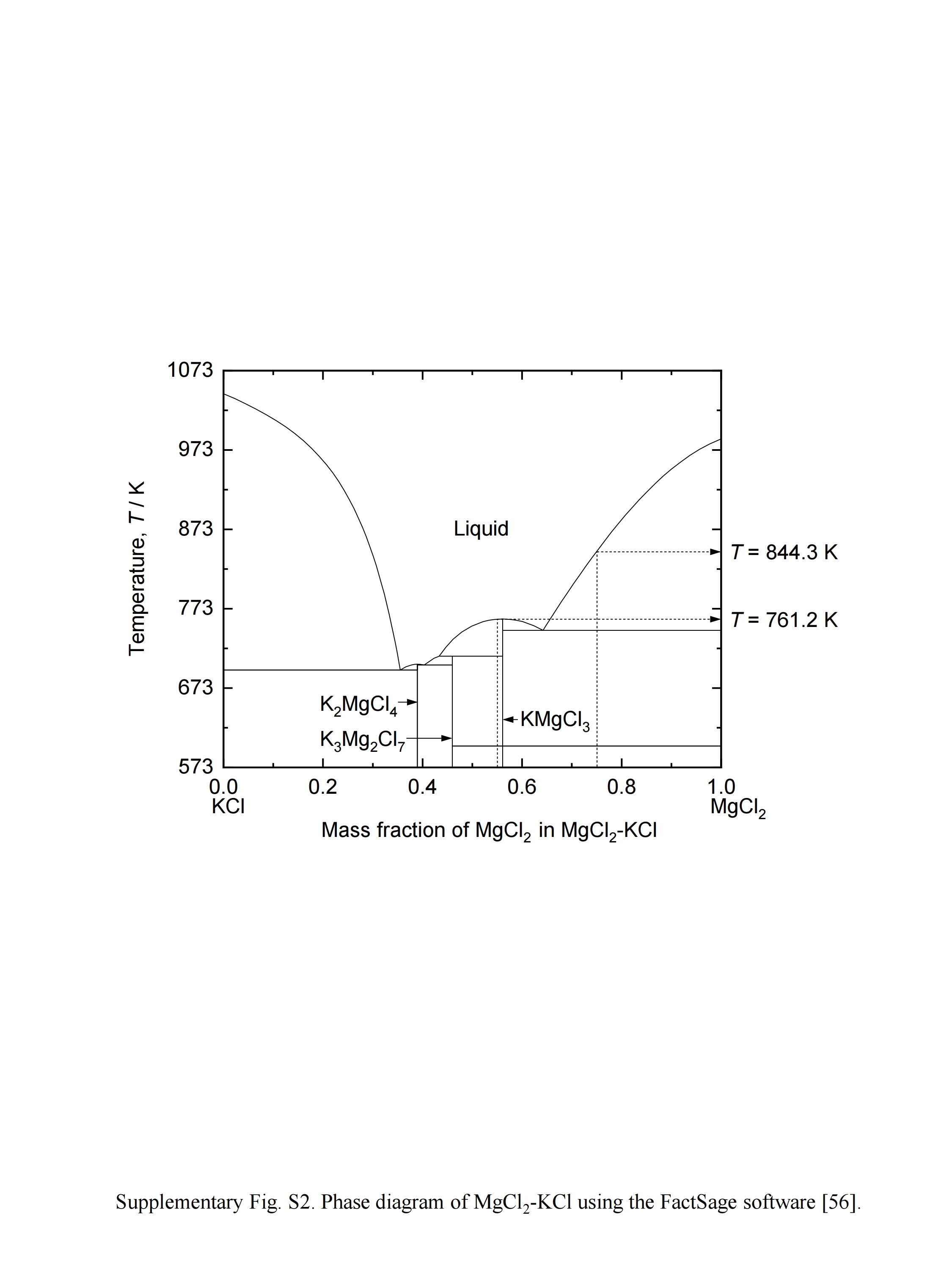

Supplement: Supplementary file 2 — Supplementary Figure S2. [file 41598_2023_50765_MOESM2_ESM.jpg]
